# Depressive and Aggressive Responses to Frustration: Development of a Questionnaire and Its Validation in a Sample of Male Alcoholics

**DOI:** 10.1155/2011/352048

**Published:** 2011-03-17

**Authors:** M. Y. Baars, M. J. Müller, B. Gallhofer, P. Netter

**Affiliations:** ^1^Department of Psychology, University of Giessen, Otto-Behaghel-Strasse 10, 35394 Giessen, Germany; ^2^Vitos Giessen-Marburg Gemeinnützige GmbH, Licher Strasse 106, 35394 Giessen, Germany; ^3^Clinic of Psychiatry, University of Giessen, Am Steg 22, 35392 Giessen, Germany

## Abstract

Since clinical and biochemical observations point to much overlap between depression and aggression, both characterised by intolerance to frustration, a questionnaire was developed to test if different patterns of depressive and aggressive reactions elicited by exposure to negative events and deprivation from expected positive ones in human and nonhuman conditions, respectively, would result in specific response patterns in depressive and aggressive persons. The questionnaire was tested for internal consistency in a pilot healthy sample and for correlations of responses with the personality factors of Aggression and Depression in 60 abstinent male alcoholics. Aggressive and depressive responses were highly correlated across all stimulus conditions, and not specifically but rather equally associated with the personality factors of Aggression and Depression, confirming the close association between these dimensions.

## 1. Introduction

Since the present paper deals with aggression and depression in the context of a psychopathological disorder, the following consideration has to be addressed as a premise.

It has already been claimed by Kretschmer [1] and Eysenck [2] that symptoms of psychiatric diseases may be observed on a milder level in nonclinical populations which suggests a continuum between disease and normal behavior. Psychologists used some of these symptoms as items to construct scales by factor analysis for specific pathology related personality traits like depression or aggression. Such scales nowadays usually form subscales of broader personality inventories like the NEO-PI-R used for assessment of the five factor model of personality. When applied to clinical samples, personality scales like those of the NEO-PI-R have been shown to be predictive of specific personality disorders [3, 4]. Scales of neuroticism, depression, and anxiety yield higher scores in depressed patients [5, 6], and scales measuring reactive or spontaneous aggression yield higher means in patients with impulse control disturbances, antisocial personality disorders [7] or alcohol dependence [8] than in nonclinical groups. Therefore, scores of depression and aggression on personality tests are conceived as models for respective psychopathological symptoms.

Depression and aggression are considered to belong to different classes of diagnoses according to psychiatric classification systems (DSM-IV and ICD-10) and to different factors in personality inventories (e.g., NEO-PI-R). Yet, there is biochemical and clinical evidence for a relationship between the two constructs.

Since the discovery of neurotransmitter abnormalities as biological markers for psychiatric disorders, a possible common basis of depressive and aggressive symptoms has been discussed in particular on the basis of serotonin [9, 10], because low 5-hydroxy-indolamino-acid (5-HIAA) levels had been discovered in the cerebrospinal fluid of violent suiciders [11] and because serotonin agonists and uptake inhibitors tend to reduce symptoms of depression [12] as well as of aggression [12–14] and because abnormal hormone responses to serotonergic challenge tests are correlated with scores on depression and impulsivity scales. Clinical evidence for overlap is, on the one hand, given by the psychoanalytic view [15, 16] that depression results from aggression turned inward against the self and, on the other hand, from the observation of depressive as well as aggressive features in patients with major depression [17, 18] as well as in alcoholics [8], where subtypes of depressed groups with and without certain aspects of aggression could be identified.

Both depression [19] and aggression [20, 21] are characterized by low tolerance to frustration which gave rise to the present investigation. The original frustration-aggression hypothesis [22] claiming that frustration always leads to aggression was revised by Miller [23] who argued that aggression is only one of the possible responses to frustration which would permit aggressive as well as depressive responses. Therefore, it may be asked if aggressive and depressive responses to frustration are also expected to share common variance like the traits, that is, if they are positively correlated or mutually exclusive. 

According to Gray's original Reinforcement Sensitivity Theory (RST) [24], the neurobiological systems BIS (behavioural inhibition system) and BAS (behavioral activation system) reflect reactivity to signals of punishment or nonreward and reactivity to signals of reward or non-punishment, respectively. Although deprivation from positive reinforcers and encounter with negative events both reflect facets of the BIS system, several psychopathological syndromes like antisocial personality disorder, depression, or drug dependence suggest that positive and negative reinforcers may differ in salience according to type of psychiatric disease. This can be derived from the observation that deficiency of reward is the primary reason for committing criminal acts in antisocial personality disorder [25, 26] and that high sensitivity to punishment is characteristic of disorders with depressive and anxiety related symptomatology [24]. So persons with antisocial personality disorders or drug dependence may react more severely when deprived from their expected rewards, while anxious-depressive persons who, according to Gray [24], are more susceptible to punishment, would be expected to feel more frustrated when being criticised or confronted with external obstacles suitable to prove their inability to handle challenges.

An additional question would be whether predominantly depressive or aggressive reactions to frustration do not only depend on the personality factors of Depression and Aggression but also on the type of frustrating condition.

A previous instrument investigating different types of responses to frustration is the projective Rosenzweig Picture Frustration Test (PFT) [27] which also focuses on responses reflecting depression associated intropunitive and aggression related extrapunitive responses, but the stimulus material only represents conditions depicting social interactions and no inanimate obstacles and, furthermore, does not distinguish between punishment and nonreward. Also the punishment subscale of the Sensitivity to Punishment and Reward Questionnaire (SPSRQ) by Torrubia et al. [28] does not address different types of punishment conditions and different types of responses.

Interpersonal disappointments or negative reactions of social partners, for instance, may induce more depressive reactions than frustrations caused by nonhuman obstacles, and conditions imposed by regulations of the police or technical failure may elicit stronger aggressive responses than frustrations deriving from personal interaction with a social partner. Therefore, this source of variance has also got to be considered when analyzing response differences to deprivation from positive and encounter with negative stimulus conditions. 

Moreover, it is known that deliberately caused frustrations will elicit stronger aggressive responses than unintentional ones [29], so this distinction has also to be taken into account for comparing different stimulus conditions. 

Therefore, it was considered to construct a questionnaire on daily frustrations (QDF) which permits to discriminate between the two facets of frustration: punishment and nonreward by depressive as well as aggressive reactions, and which relates to human as well as to nonhuman frustrating conditions (study 1).

Since intolerance to frustration is very pronounced in drug addicts, it was expected to be also particularly high in alcoholics. Furthermore, as outlined above, depressive and aggressive personality traits are expected to be both observed in alcoholics [8]. This is supported by Cloninger's theory [30] that alcoholics represent two different types of alcoholism: type 1 is characterized by later onset and a predominance of problem drinkers frequently characterized by depression, whereas type 2 shows high heritability, early onset, and is associated with antisocial personality. So, it is expected that in a sample of alcoholics both highly depressive and highly aggressive personality traits will be observed.

**Study 1**

The following questions have to be answered in this study.

1. Do the scales of the QDF reveal internal consistency?
2. Is there a difference in means of responses to the items representing deprivation from positive reinforcers (pos) and those representing the encounter with negative events (neg), and does this difference depend on the type of stimulus condition (intentional/unintentional and human or nonhuman source)?
3. Is there a positive, zero, or negative correlation between depressive and aggressive responses to the same set of item categories?

## 2. Methods

### 2.1. Construction of the QDF

A total of 32 frustrating events, including topics such as partnership, money, work, and social contacts, had been collected and presented in 2-3 short sentences each. 16 of them represent deprivation from positive reinforcers (rewards), and 16 refer to the confrontation with negative reinforcers (punishments). Within each set of stimuli, 8 events are caused by external, nonhuman faults, 8 by humans in a social situation. The 8 frustrations elicited by humans are divided into 4 situations, each in which a person deliberately (h++) or unintentionally (h+) causes the frustration. The sum of h++ and h+ is labelled H. The events caused by nonhuman faults are labelled NH. This results in four item categories named posNH, posH, negNH, and negH, or in 8 categories including the additional groups of items posh+, posh++, negh+, and negh++. 

Each situation is followed by 6 distinct emotional reactions, which have to be marked on a 0 to 10 point Likert scale of 0 = “does not apply to me at all” to 10 = “applies to me very much”. This results in altogether 32 × 6 = 192 items. The 6 responses always consist of two reactions labeled as depressive, aggressive, and neutral each. The number of the particular reaction is attached to the label of the scale as 1–6. This yields 8 × 6 = 48 scales altogether.

The full set of 32 situations of the questionnaire, translated from the original German version in the version given to males, is attached in the appendix. Sample situations for each of the categories described above are given below with category labels in bold letters.

(2) You are queuing at a box office of a cinema with the intention to see a movie premiere that you have been waiting for since a long time. Finally, it is your turn, but you are informed that all tickets are sold out **(posNH)**.
(4) You have been looking forward very much to a weekend trip with your girl friend/partner. Since a relative of hers has become sick and asks her for help, she has to cancel the trip **(posh+)**.
(7) You have made every effort to prepare a pleasant birthday party for your friends. Unfortunately, most of them are in a bad mood and therefore all of them leave the party very early, giving different excuses **(posh++)**.
(16) Just for a moment, you are leaving your flat without taking a key with you while the door remains open. But a heavy blast shuts the door and you are locked out **(negNH)**.
(17) You are preparing a sophisticated meal while you receive a telephone call from a friend. You are so preoccupied with your conversation that in the kitchen the food is burning **(negh+)**.
(20) At work you always give your very best and you are also very conscientious. Yet, your boss always criticizes you for working too slowly or making too many mistakes **(negh++)**.

Reactions following each of the 32 situations.

1. You tell yourself: “This always happens only to me” **(depressive)**.
2. You consider how to make the best of it **(neutral)**.
3. You become angry and start swearing **(aggressive)**.
4. You think: “So what, such things just happen” **(neutral)**.
5. You blame yourself for this event **(depressive)**.
6. You blame everybody else **(aggressive)**.

For example, “item 20.4” means reaction 4 (You think: “So what, such things just happen.”) as a response to item 20.

### 2.2. Sample and Data Collection

A sample of 50 healthy German persons (males *n* = 17; females *n* = 33; age: median = 29 years; range = 20–70) was recruited (a) among undergraduate Psychology students from the University of Giessen, Germany (*n* = 35). The experimenter informed the undergraduates before they entered the auditorium to a plenary Psychology lecture. Since Psychology students in Giessen have to prove that they have served as experimental subjects for altogether 30 hours, only undergraduates participated who still needed additional hours for their records. (b) These participants were supplemented by acquaintances of the experimenter and their relatives (*n* = 15) who were personally approached and received a bar of chocolate as a reward for participating. All subjects were instructed to fill in the QDF which for reasons of data protection was only labeled by a number and had to be returned anonymously in a closed envelope to a box in the secretary's office or by mail. The study was approved by the ethics committee of the Medical Faculty of the University of Giessen, Giessen, Germany. 

### 2.3. Statistical Evaluation

For reliability analysis, item-total-correlations and Cronbach's Alpha were computed for each of the 48 scales (Table 1). After applying the Levine test in order to test for homogeneity of variances, *t*-tests for independent groups were used for testing differences in means between males and females and and *t*-tests for dependent samples were applied for testing differences between means of corresponding responses given to items representing deprivation from positive and application of negative reinforcers within human and nonhuman categories. After having tested the scales for normal distribution of item responses by the Kolmogorov-Smirnov test, Pearson correlations were computed for analyzing the relationships between corresponding responses to categories of nonreward and punishment and between aggressive and depressive responses. These correlations are merely reported on a descriptive level using an alpha level of  .05 without alpha adjustment. Bonferroni corrections of significance levels were, however, performed for the *t*-tests. All statistical analyses were performed by SPSS version 11.5.

## 3. Results

In order to test if gender could operate as a confounder, sex differences were tested for all 48 scales. They were found to be significant only in scales posh3++ and posh4++ (Table 1), that is, males feel more anger and females are more relaxed or forgiving in conditions of being deliberately deprived from a positive reinforcement by another person. Since these were the only differences observed between the male and female sample and since the male sample was very small anyhow, further evaluations will not take gender into account. 

### 3.1. Internal Consistency of Scales (Question  1)

Table 2 shows the reliability analyses of the 8 × 6 = 48  QDF scales. 

For most of the scales, Cronbach's Alpha reveals acceptable internal consistencies. For this analysis, also items with corrected item-total-correlations below *r* = .30 were retained in order to keep the parallel structure of the questionnaire and for considering face validity. They will, however, be eliminated for the validation of the questionnaire in a clinical sample. It is obvious that the shorter 4 item scales show lower reliabilities than the longer ones. 

### 3.2. Comparison between Deprivation from Positive and Encounter with Negative Reinforcements according to Stimulus Conditions (Question  2)

**Figure 1:**
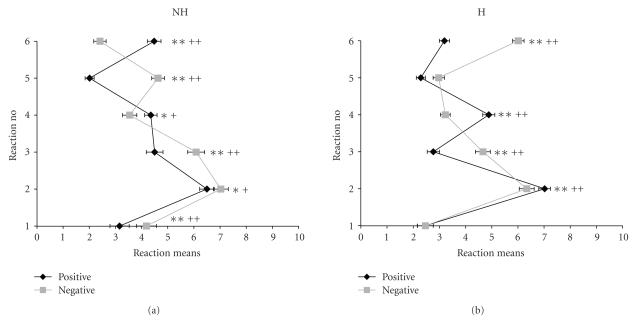
mean reactions + SEM to withdrawal of positive reinforcers (pos) and encounter with negative reinforcers (neg) in nonhuman (NH, (a)) and human (H, (b)) conditions of frustration (1–6 see responses to QDF scales in Section 2.1., 1 + 5 = depressive, 3 + 6 = aggressive, and 2 + 4 = indifferent responses; *P < .05; **P < .01 before Bonferroni adjustment; ^+^ *P* < .05; ^++^ *P* < .01 after Bonferroni adjustment of significance level).

Means of responses to the two types of frustration, separated according to nonhuman and human conditions are depicted in Figure 1.

Nearly all *t*-tests performed for comparisons between means of the “positive” and “negative” frustration scales within each of the corresponding reactions for nonhuman as well as for human sources of frustration revealed significant differences. 

The most prominent finding is that in nonhuman conditions of frustration (NH) people tend to blame others (response 6) more when deprived from anticipation of reward (pos) than when frustrated by obstacles (neg), whereas respective conditions caused by humans show the opposite pattern. This difference remains significant on the 1% level of significance after Bonferroni correction. In condition NH, blaming oneself as opposed to blaming others is more pronounced when confronted with being blamed or insulted (neg) than when deprived from reward (pos), a difference which also remains significant after alpha adjustment.

**Figure 2:**
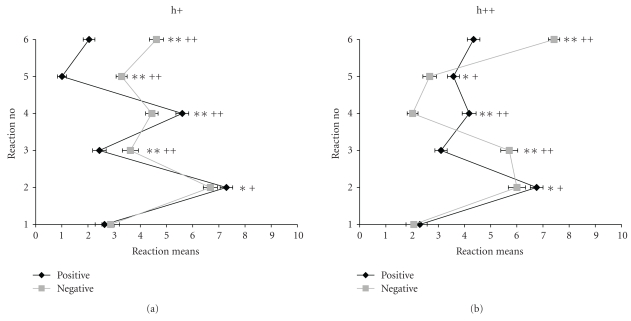
mean reactions + SEM to withdrawal of positive reinforcers (pos) and encounter with negative reinforcers (neg) in conditions of unintentional (h+, (a)) and deliberate (h++, (b)) frustration by humans (*P < .05; **P < .01 before Bonferroni correction; ^+^ *P* < .05; ^++^ *P* < .01 after Bonferroni adjustment of significance levels).

Reactions to intentional and unintentional frustration caused by social partners are depicted in Figure 2.

Comparing the profiles of the corresponding scales of “positive” and “negative” frustrations within the categories of deliberate (h++, Figure 2(a)) and unintentional human frustrations (h+, Figure 2(b)), it can be seen that the course of the diagrams for “positive” and “negative” frustrations are very similar for unintentionally as well as for deliberately elicited frustrations. However, they differ markedly for intensity of reactions 3 (becoming angry) and 6 (blame everybody else) in the frustrations caused deliberately, that is, intentionally inflicted aversive social acts elicit more aggressive responses than denial of expected rewards. Since differences between the h+ and h++ scales are not very pronounced with respect to reaction profiles and since, furthermore, these scales only consist of 4 items each, which reduces the internal consistencies (Table 2), the scales h+ and h++ will no longer be analyzed separately, but as a combined scale H. 

### 3.3. Correlations between Aggressive and Depressive Responses (Question 3)

All intercorrelations among all of the 48 scales are listed in Tables 3, 4, 5, 6, 7, and 8 which provide the basis for answering question 3. Regularly, the scales of reactions 1, 3, 5, and 6 show significant positive intercorrelations, demonstrating that persons who respond by aggressive reactions (3 and 6) also tend to react in a depressive way (responses 1 and 5). Similarly, reaction scales 2 and 4, the indifferent responses, are positively correlated with each other, but between the set of scales 1, 3, 5, 6, and the two scales 2 and 4, the associations are negative or nonsignificant. This means that relaxed responses (4) and active coping (2) are negatively related or unrelated to the emotional depressive and aggressive reactions. This is observed across all categories of situations, as well as within categories.

## 4. Conclusions (Study 1)

The results reveal that although fair internal consistencies for the reaction scales to the four major categories, posNH, negNH, posH, and negH, have been achieved, some reactions are inappropriate for certain situations and have to be eliminated due to their low item-total-correlations (question  1). Moreover, although the situations can be significantly discriminated into frustrations due to deprivation from reward and application of punishment (“positive” and “negative frustrations) by the intensities of emotional reactions (question  2), the types of depressive and aggressive reactions do not form opposite emotional responses but are positively related (question  3). They are not highly specific for the types of stimulus classes and are both negatively correlated or unrelated to being relaxed or inclined to active coping.

The results reveal that although fair internal consistencies for the reaction scales to the four major categories posNH, negNH, posH, and negH have been achieved, some reactions are inappropriate for certain situations and have to be eliminated due to their low item-total-correlations (question  1). Moreover, although the situations can be significantly discriminated into frustrations due to deprivation from reward and application of punishment (positive and negative frustrations) by the intensities of emotional reactions (question  2), the types of depressive and aggressive reactions do not form opposite emotional responses but are positively related (question  3). They are not highly specific for the types of stimulus classes and are both negatively correlated or unrelated to being relaxed or inclined to active coping.

**Study 2**

Questions to be tested in this study are as follows.

1. Are correlations between the trait of depression higher with the depressive QDF responses than with the aggressive ones and is aggression more correlated to the aggressive QDF responses than to the depressive ones?
2. Do responses to nonreward (pos) show stronger associations with the trait of aggression than responses to punishment (neg) and do responses to punishment (neg) show stronger correlations with the trait of depression than responses to nonreward (pos)?
3. Do the results reveal higher responses to human than to nonhuman conditions of the QDF scales in depressive alcoholics and is this relationship absent in aggressive alcoholics?

## 5. Methods

### 5.1. Sample and Data Collection 

The sample of *n* = 60 patients (age: mean = 47.93; SD = 9,00; range = 27–69) included in study 2 had to fulfill the following criteria: alcohol abuse as defined by the ICD-10 code F10.2 according to the WHO, diagnosed by an experienced psychiatrist, male gender, age > 18 years, no additional substance dependence, sufficient knowledge of the German language. Patients who additionally suffered either from schizophrenia, schizotypal, or delusional disorder or from bipolar affective disorder according to the WHO ICD-10 classification were excluded. Patients were recruited on the one hand from two German psychiatric hospitals (University Hospital Giessen-Marburg and Vitos Hospital Giessen), after acute withdrawal, and on the other hand from two outpatient institutions for psychotherapy of alcohol addiction after withdrawal in one of the two psychiatric hospitals. They were asked to give informed consent and were rewarded by 20 Euro after completion of the session. The study was approved by the ethics committee of the Medical Faculty of Giessen University, Giessen, Germany.

### 5.2. Questionnaires

The patients were asked to fill in the following personality questionnaires:

1. the newly constructed Questionnaire of Daily Frustrations QDF [31],
2. the Questionnaire on Factors of Aggression FAF(Hampel and Selg, 1975) [32],
3. the General Scale on Depression ADS (Hautzinger and Bailer, 1993) [33].

Since aggression is closely related to impulsivity and depression to anxiety, the following questionnaires were added for increasing discriminant construct validity:

(4) Eysenck's Impulsivity scale I7 (Eysenck and Eysenck, 1978) [34],
(5) the Sensation Seeking Scales SSS-V by Zuckerman et al. (1978) [35],
(6) the Interaction Anxiety Questionnaire IAF by Becker (1997) [36],
(7) the Sensitivity to Punishment and Reward Questionnaire SPSRQ by Torrubia et al. (2001) [28],
(8) the Impulsivity Scales BIS-11 by Barratt (Patton et al., 1995) [37].

### 5.3. Statistical Evaluation

A principal component analysis with varimax rotation was performed on these questionnaire scales in order to identify broader factors of depression and aggression related traits. Two major factors emerged representing Depression and Aggression beside two other factors identified as Impulsivity and Anxiety. The factor of Depression was composed of the General Depression Scale ADS (loadings added in parenthesis: *a* = .910) and the FAF subscale 4 = accusing oneself (*a* = .828), and the factor of Aggression comprised the FAF subscales 1 = spontaneous aggression (*a* = .827) and 2 = reactive aggression (*a* = .912). Factor scores for each participant were formed by adding the *z*-transformed values of the respective scales comprising each of the two factors.

For reliability analysis, item-total-correlations and Cronbach's Alpha were computed for each of the 6 × 8 = 48 QDF scales. Items with corrected item-total-correlations below *r* = .30 were eliminated (items deleted: 1.1; 2.4; 3.6; 4.6; 8.5; 8.6; 10.6; 11.5; 14.5; 14.6; 15.5; 16.6; 17.5; 17.6; 18.6; 19.5; 19.6; 20.6; 21.5; 21.6; 22.6; 23.5; 24.5; 24.6; 25.5; 26.6; 28.5; 32.5). In order to keep the scales comparable after elimination of several items, response scales were divided by the number of remaining items so that scores ranged between 1 and 10 on each of the 48 Likert scales. 

Pearson correlations were computed between the QDF response scales and the personality clusters representing the traits of Depression and Aggression. Differences between correlation coefficients were tested by *z*-tests (*t*-tests applied to *z*-transformed correlation coefficients). Bonferroni adjustment of significance levels was performed separately for each set of 6 correlations (6 response scales) with each of the two personality traits. All statistical analyses were performed by the SPSS Version 11.5

## 6. Results

### 6.1. Correlations between Trait and State Variables of Depression and Aggression (Question 1)

**Figure 3:**
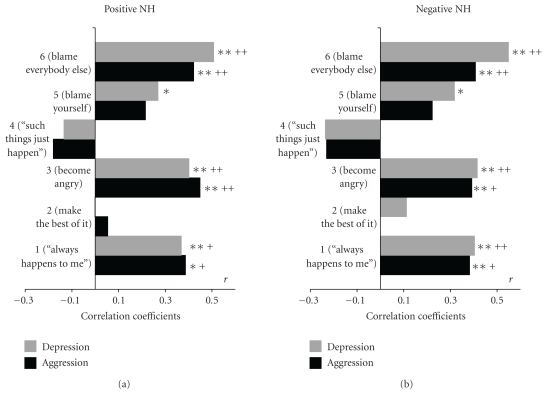
correlations of factors Depression and Aggression with QDF scales for nonhuman conditions; withdrawal of positive reinforcers: posNH, (a); encounter with negative reinforcers: negNH, (b) (*P < .05; **P < .01; ^+^ *P* < .05; ^++^ *P* < .01 after Bonferroni correction).

**Figure 4:**
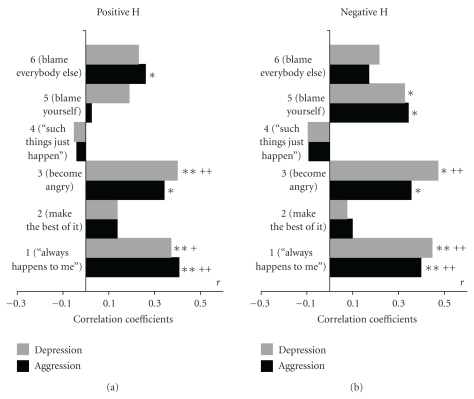
correlations of factors Depression and Aggression with QDF scales for human conditions; withdrawal from positive reinforcers: posH, (a); encounter with negative reinforcers: negH, (b) (*P < .05; **P < .01; ^+^ *P* < .05; ^++^ *P* < .01 after Bonferroni correction).

For answering questions 1, 2, and 3, Figures 3 and 4 depict the correlations of the QDF scales with the personality factors Depression and Aggression mentioned above for nonhuman sources of frustration (Figure 3) and for human sources of frustration (Figure 4), each depicted for all 6 response scales to withdrawal from positive reinforcers (pos, (a)) and application of negative reinforcers (neg, (b)).

Significant correlations between responses, in particular responses 1 (happens only to me) and 3 (get angry) with both, Depression and Aggression, can be found within all four conditions (Figures 3 and 4 left and right panel). The hypothesis would suggest that Depression should show higher correlations with the depressive responses 1 and 5 than Aggression, and Aggression should be more intensively related to responses 3 and 6 than Depression. Regarding the significant correlation coefficients, this is neither the case for the negative reinforcement condition nor for the condition of withdrawal from rewards in either human (H, Figure 3) or nonhuman (NH, Figure 4) sources of frustration. Even in the few instances in which pairs of correlations according to inspection would support the hypothesis (Figure 3 response 3, pos NH, and response 5 both, pos and neg NH), no significant differences between the Aggression and Depression coefficients can be proven by *z*-tests. Also on a descriptive level from the 6 significant correlation coefficients between depressive responses 1 or 5 and Depression, three showed higher correlations with Depression and three with Aggression. Even more surprising was that out of the 6 significant correlations of the aggressive responses 3 and 6 with the personality factors, five showed higher correlations with Depression than with Aggression indicating that Depression seems to be more responsible for both types of responses to frustration than Aggression, and that aggressives do not seem to be more inclined to respond by aggressive reactions than depressives. It seems that neither Depressive nor Aggressive types prefer their trait congruent reactions. So, the hypothesis of specific trait-state relationships has to be rejected. 

### 6.2. The Relations between Punishment and Depression versus Nonreward and Aggression (Question  2)

For testing the hypothesis derived from Gray's theory that depressives are more sensitive to punishment than to withdrawal of reward corresponding correlations of Depression and Aggression, respectively, with the QDF response items of the left (pos) and right panel (neg) of each figure compared by *z*-tests. Although no significant differences between corresponding Items of the left and right panel could be detected, on a descriptive level correlations with Depression with each of the relevant responses 1, 3, 5, 6 were higher for responses to negative reinforcers than for denial of positive events, particularly in the nonhuman conditions of frustration (Figure 3), so that this part of the hypothesis gets some support. For Aggression, no clear pattern emerged since only half of the correlations with the responses were higher for denial of positive reinforcers than for encounter with negative events. Surprisingly, aggressives even tended to accuse themselves (reaction 5) and not the other person (reaction 6) when being insulted or attacked by other persons (Figure 4(b), negH). Taken together, the situation by personality interaction expected for the two stimulus conditions according to question 2 could not be found in our data. 

### 6.3. Differences in Correlations between Frustrations Caused by Humans and NonHuman Conditions (Question  3)

It was hypothesized that depressives as opposed to aggressives might be more sensitive to frustrations caused by humans than to frustrations by external inanimate obstacles. Although this seems to apply to getting angry (reaction 3) which was higher with Depression in frustrations caused by humans than in the nonhuman conditions, statistical comparisons between correlations of Depression with corresponding responses to person induced as opposed to inanimate frustrations, did not yield significant differences by *z*-tests. Rather, it becomes evident that in particular reaction 6 (blaming others) is less associated with both personality factors Depression and Aggression when elicited by frustrations caused by humans (Figure 3) than by inanimate frustrations (Figure 4). So, there is no convincing evidence for a specific affinity of depressives to frustrations by humans.

Since the traits of Aggression and Depression are positively correlated with each other (*r* = .374), partial correlations with Depression were computed controlling for Aggression and partial correlations with Aggression partialling out Depression (see Table 9). 

All correlations were lower than the original ones, but mostly still significant, although partly only on the  .05 level. This demonstrates that in spite of some common variance each of the two constructs contributes special variance to the response variables which were significantly related to the traits.

In reply to question 1, partial correlations between depressive responses 1 and 5 and aggressive responses 3 and 6 on the one hand and the corresponding personality factors on the other were compared across all stimulus conditions on a descriptive level. No clear relationship between corresponding trait and state variables could be observed, since from the eight correlation coefficients between depressive responses 1 or 5 and Depression, three showed higher correlations with Depression and three with Aggression (two were not significant). Even more surprising was that out of the 6 significant correlations of the aggressive responses 3 and 6 with the personality factors, five showed higher correlations with Depression than with Aggression indicating that Depression seems to be more responsible for both types of responses to frustration than Aggression, and that aggressives do not seem to be more inclined to respond by aggressive reactions than depressives. This confirms the high correlation between aggressive and depressive reactions across all conditions observed in study 1.

In response to question 2, the hypothesis derived from Gray's theory [16] that depressives are more sensitive to punishment than to withdrawal of reward can partly be confirmed, since for each of the relevant responses 1, 3, 5, 6 corresponding correlations with Depression were higher for responses to negative reinforcers than for denial of positive events, particularly in the nonhuman conditions of frustration (Figure 3), so that this part of the hypothesis can be confirmed although none of the differences reach significance. For Aggression no clear pattern emerged, since only half of the correlations with the responses were higher for denial of positive reinforcers than for encounter with negative events. Surprisingly, aggressives even tended to accuse themselves (reaction 5) and not the other person (reaction 6) when being insulted or attacked (Figure 4(b), negH). Taken together, the situation by personality interaction expected for the two stimulus conditions according to question 2 could not be found in our data.

Finally, with respect to question 3, patterns of correlations between the personality factors and responses to corresponding human and nonhuman conditions of frustrations were compared (Figure 3 versus Figure 4). The patterns for correlations with Depression and Aggression were fairly similar: for reaction 6 (blame others), the correlations were always higher in conditions of nonhuman as compared to human sources of frustration, the “negative” conditions yielding clearly higher differences between coefficients than the “positive” frustrations. Conversely, reaction 1 (only happens to me) always yielded higher correlations with both personality factors in human than in nonhuman conditions. For the condition of blaming oneself (reaction 5) correlations with both Depression and Aggression were higher in nonhuman conditions than in those caused by humans in the situations of deprivation from reward, but vice versa when confronted with negative reinforcement. The only clearly specific response suitable to distinguish between the depressive and aggressive personality factor was getting angry (reaction 3) which was more significantly correlated with Aggression as a response to inanimate obstacles both when deprived from reward and when confronted with negative events, and was higher with Depression in all frustrations caused by humans than in the nonhuman conditions. So, only this latter result might be a weak hint that depressives tend to be more frustrated by social interactions than by external mischief and that an opposite reaction is characteristic for aggressives. 

## 7. Discussion

In study 1, it was tried to construct a questionnaire on reactions to daily frustrations (QDF) suitable to distinguish between frustrations caused by withdrawal of positive reinforcers (nonreward) and by infliction of negative reinforcers (punishment) and to distinguish between human and nonhuman sources of frustration by different response patterns of depressive, indifferent, and aggressive reaction categories to each item. The resulting 2 (positive/negative reinforcers) × 2 (human/nonhuman condition of frustration) = 4 resulting categories in the pilot study revealed that it is worthwhile to distinguish between these stimulus categories, because reactions were usually more pronounced when encountering negative reinforcements than when deprived from expected positive stimuli. In particular, the reaction of blaming others was suitable to distinguish between punishment and nonreward and this difference was reversed for the human and nonhuman source of frustration. It became evident, however, that in spite of good internal consistencies of the scales comprising the four item categories, some types of reactions cannot be equally well applied to conditions in which human or nonhuman sources of frustration are involved. This also became evident in the clinical sample so that some reactions to single items had to be eliminated due to low part-whole correlations with the scale scores before further analyses with the QDF scales in study 2. 

Furthermore, it was remarkable that aggressive reaction items like getting angry and those of a depressive nature like self-pity were very highly correlated in all stimulus conditions confirming that the revised frustration aggression theory [23] does not imply alternative responses to frustration but that both aggressive and depressive reactions may occur in the same person in the same condition. This also applies to the stronger responses of blaming oneself and blaming others which may also be present simultaneously but in different intensities depending on the type of frustration.

It must be admitted, however, that the sample size of the pilot study on which scale construction was based is extremely small und requires replication in larger samples representing broader distributions of demographic variables. It is hoped that providing the test in the internet will help to test its suitability in different groups of healthy as well as clinical samples.

We are also aware that the distribution of age in that sample was skewed and that motivation for participating was different for students and nonstudents and confounded by age. We therefore computed correlations between age and all the 48 QDF scales. Only two of them reached a significance level of *P* < .05 which is compatible with error.

The evaluations performed in study 2 were based on the concept that depression and aggression as measured by personality tests can be understood as continua ranging from normal personality to psychopathology and may, therefore, serve as models for studying depression and aggression in clinical samples.

The first aim of the study was to answer the question, if the depressive and aggressive responses to frustrations are mediated by the personality traits of depression and aggression, respectively. Instead of single scales, factors derived from several scales measuring depression and aggression were applied in order to increase validity. Depressive and aggressive responses to frustrating situations did not turn out to be specific for the respective traits of depression and aggression, but rather showed similar correlation patterns with the two dimensions. This could be assumed to be due to the fact that depression and aggression are frequently combined in alcoholics [8, 30] and might, therefore, also be responsible for the fairly high correlation between the two traits and their overlapping correlations with depressive and aggressive QDF response scales. However, in the healthy sample of study 1 the depressive and aggressive responses to the QDF were also highly correlated and, furthermore, similar correlation patterns with QDF scales were still observed after partialling out the trait score of Depression and Aggression, respectively. So, our data seem to confirm clinical observations of the relationship between aggression and suicidality [11, 18] or the comorbidity of depression and aggression, for example, in Attention Deficit Hyperactivity Disorder (DSM-IV) and would fit the idea of disturbances of the serotonin system as a common underlying biochemical basis of aggressive and depressive symptoms [10]. The finding also confirms the observation that the subscales of outward aggression and self-accusation in the FAF are positively correlated [32] which seems to corroborate the old psychoanalytic view that depressive symptoms of guilt feelings and self-accusation reflect aggression turned inward [15, 16]. So, it must be assumed that the data confirm the theory that Depression and Aggression are complementary components of a psychological disturbance as suggested already by neurochemical findings [9, 11]. 

The second aim was to test if the two aspects of the “punishment” system (withdrawal of reward “pos” and infliction of punishment “neg”) according to Gray [24] can be separated by testing their relation to the dimensions of aggression and depression. Our hypothesis was that reactions to frustration from nonreward could be deduced from reports of increased reward sensitivity in certain disorders like impulse control disturbances and substance abuse. Although the two conditions did elicit partly different responses, as shown by differences of means in study 1, it can be concluded from high correlations between corresponding responses to the two conditions that it is hard to separate them. This was the reason that Gray always regarded the two aspects as identical. But yet it seems worthwhile to follow the idea of separable aspects of frustration by improving the questionnaire and applying it in further clinical groups.

The third question related to the discrimination between social and inanimate frustrations was not very much related to the dimensions investigated in this study but was suitable for characterizing symptoms of the alcohol history like resistance to therapy as shown in a different evaluation of the present study [31].

It must be considered that the only moderate tendency to express anger or to accuse others when frustrated by humans as compared to nonhuman conditions may be a particular feature of alcoholics most of whom have agreed to engage in psychotherapy and probably do not dare to express aggressive thoughts in social contexts being in a clinical setting. Patients high on aggression scores even tended to accuse themselves when attacked or insulted by another person (Figure 4(b)). 

The limitation of this study is, of course in addition to the fairly small number of cases, that the diagnosis of alcoholism was only based on ICD-10 criteria obtained by different psychiatrists. The only common feature was that all patients had undergone detoxification in a psychiatric hospital. Generalizability of results is furthermore limited by the fact that we had only male patients since just the dimensions of aggression and depression differ widely in their correlational context between males and females. 

## 8. Conclusions (Study 2)

We have constructed a symmetrically organized questionnaire on frustrations (QDF) which permits to assess different reactions to different situations organized according to the two principals of source and type of frustrations. The new aspect contributing to research in frustration is that different responses have been found not to be alternatives varying between persons or within persons across situations as conceived by Miller in the revised frustration-aggression theory but could be shown to occur simultaneously in the same person. We were also able to contribute to depression research by demonstrating a very close relationship with aggression on the level of traits as well as on the level of states suitable to remind psychiatrists and psychologists when performing their clinical assessment with patients, that seemingly contradictory features like aggression and depression often have to be considered and diagnosed simultaneously in the same patient. It is intended to try the questionnaire in particular in other clinical groups characterized by depression and/or aggression like borderline personality disorder, ADHD, bipolar disorder, and different subgroups of schizophrenics, in order to test, if the QDF may be more suitable to discriminate persons according to responses to punishment and nonreward than it was the case with alcoholics. 

## Figures and Tables

**Table 1:** Gender differences in the subscales of the QDF (means, SD, SEM, and significance of differences *P*).

Scales	Mean		SD		SEM		*P*	Scales	Mean		SD		SEM		*P*
	Male	Female	Male	Female	Male	Female			Male	Female	Male	Female	Male	Female	
posNH1	2.57	3.46	2.32	2.71	0.56	0.47		negNH1	3.27	4.65	2.28	2.82	0.55	0.49	
posNH2	6.80	6.34	2.16	1.87	0.52	0.33		negNH2	7.08	7.00	2.31	1.89	0.56	0.33	
posNH3	4.15	4.68	2.11	2.35	0.51	0.41		negNH3	5.37	6.46	2.19	2.28	0.53	0.40	
posNH4	4.71	4.17	1.66	1.69	0.40	0.29		negNH4	4.23	3.19	1.97	1.85	0.48	0.32	
posNH5	2.14	1.95	1.23	1.23	0.30	0.21		negNH5	4.51	4.69	1.20	1.96	0.29	0.34	
posNH6	4.38	4.53	1.88	1.87	0.46	0.33		negNH6	2.32	2.45	1.23	1.88	0.30	0.33	
posh+1	1.91	3.00	2.07	2.67	0.50	0.46		negh+1	2.43	3.09	1.98	2.54	0.48	0.44	
posh+2	7.07	7.40	1.98	1.55	0.48	0.27		negh+2	6.53	6.75	2.16	1.80	0.52	0.31	
posh+3	1.94	2.69	1.66	1.89	0.40	0.33		negh+3	3.24	3.82	2.02	2.23	0.49	0.39	
posh+4	6.06	5.37	1.96	1.59	0.47	0.28		negh+4	4.75	4.28	1.77	1.64	0.43	0.29	
posh+5	1.13	0.94	1.23	1.22	0.30	0.21		negh+5	3.16	3.35	1.14	1.66	0.28	0.29	
posh+6	2.09	2.02	1.65	1.57	0.40	0.27		negh+6	4.50	4.67	2.08	1.82	0.51	0.32	
posh++1	1.82	2.54	1.61	2.14	0.39	0.37		negh++1	1.47	2.37	1.67	2.25	0.40	0.39	
posh++2	6.69	6.80	1.80	1.70	0.44	0.30		negh++2	6.24	5.89	2.58	2.19	0.63	0.38	
posh++3	2.38	3.48	1.25	1.74	0.30	0.30	*	negh++3	5.53	5.80	2.18	2.37	0.53	0.41	
posh++4	4.97	3.77	1.90	1.76	0.46	0.31	*	negh++4	2.07	1.99	1.45	1.49	0.35	0.26	
posh++5	3.63	3.55	1.46	1.72	0.35	0.30		negh++5	2.28	2.87	1.59	1.93	0.39	0.34	
posh++6	4.10	4.48	2.05	1.50	0.50	0.26		negh++6	7.41	7.43	1.62	1.48	0.39	0.26	

*P < .05; pos/neg = withdrawal of positive/application of negative reinforcers; NH/H = nonhuman/human sources of frustration, h+/h++ unintentional/deliberate frustration by humans; numbers 1–6 see reactions 1–6 to QDF scales.

**Table 2:** Cronbach's alpha of the QDF subscales “positive” and “negative” (legend, see Table 1).

Scales	Alpha	Scales	Alpha
posNH1	.9441	negNH1	.9387
posNH2	.8473	negNH2	.9000
posNH3	.8882	negNH3	.9215
posNH4	.7206	negNH4	.8764
posNH5	.5863	negNH5	.7432
posNH6	.7596	negNH6	.8192

posh+1	.8601	negh+1	.8205
posh+2	.7065	negh+2	.7389
posh+3	.6580	negh+3	.7122
posh+4	.4505	negh+4	.4931
posh+5	.6574	negh+5	.4453
posh+6	.4472	negh+6	.4882

posh++1	.7678	negh++1	.8645
posh++2	.5079	negh++2	.7667
posh++3	.6381	negh++3	.7836
posh++4	.6712	negh++4	.5507
posh++5	.4393	negh++5	.6273
posh++6	.5742	negh++6	.4716

posH1	.9014	negH1	.9104
posH2	.7850	negH2	.8662
posH3	.8189	negH3	.8334
posH4	.7473	negH4	.6379
posH5	.6693	negH5	.7439
posH6	.6573	negH6	.6991

**Table 3:** Intercorrelations between the reactions to scales of withdrawal from positive and encounter with negative reinforcers of the QDF.

	posNH1	posNH2	posNH3	posNH4	posNH5	posNH6	posh+1	posh+2	posh+3	posh+4	posh+5	posh+6	posh++1	posh++2	posh++3	posh++4	posh++5	posh++6
posNH1	**1**						.881**		.441**	−.321*	.301*	.453**	.791**		.430**	−.302*	.410**	.295*
posNH2		**1**						.724**		.566**				.679**		.429**		
posNH3	.502**		**1**				.464**		.733**	−.254		.348*	.358*		.707**	−.310*	.294*	.363**
posNH4	−.298*	.430**	−.349*	**1**			−.293*	.321*		.625**			−.239	.386**		.712**		
posNH5	.392**		.310*		**1**		.401**	−.274		−.271	.507**	.471**	.345*				.507**	
posNH6	.580**		.330*	−.334*	.466**	**1**	.512**	−.296*	.276	−.359*	.269	.665**	.422**	−.352*	.27	−.388**	.283*	.477**

posh+1	.881**		.464**	−.293*	.401**	.512**	**1**						.828**		.557**	−.336*	.445**	.299*
posh+2		.724**		.321*	−.274	−.296*		**1**						.743**		.405**		
posh+3	.441**		.733**			.276	.594**		**1**				.366**		.812**	−.299*		.330*
posh+4	−.321*	.566**	−.254	.625**	−.271	−.359*	−.372**	.547**	−.291*	**1**			−.315*	.541**	−.249	.648**		
posh+5	.301*				.507**	.269	.417**	−.301*	.303*	−.282*	**1**		.425**	−.337*	.254		.496**	
posh+6	.453**		.348*		.471**	.665**	.518**	−.323*	.434**	−.283*	.438**	**1**	.487**	−.353*	.362**	−.249	.393**	.445**

posh++1	.791**		.358*	−.239	.345*	.422**	.828**		.366**	−.315*	.425**	.487**	**1**					
posh++2		.679**		.386**		−.352*		.743**		.541**	−.337*	−.353*		**1**				
posh++3	.430**		.707**			.27	.557**		.812**	−.249	.254	.362**	.355*		**1**			
posh++4	−.302*	.429**	−.310*	.712**		−.388**	−.336*	.405**	−.299+	.648**		−.249		.498**	−.339*	**1**		
posh++5	.410**		.294*		.507**	.283*	.445**				.496**	.393**	.448**			−.319*	**1**	
posh++6	.295*		.363**			.477**	.299*		.330+			.445**	.335*	−.286*	.425**	−.**257**		**1**

*P* < .1; *P < .05; **P < .01.

**Table 4:** Intercorrelations between the reactions to scales of withdrawal from positive and encounter with negative reinforcers of the QDF.

	posNH1	posNH2	posNH3	posNH4	posNH5	posNH6	posh+1	posh+2	posh+3	posh+4	posh+5	posh+6	posh++1	posh++2	posh++3	posh++4	posh++5	posh++6
negNH1	.878**		.484**	−.356*	.478**	.563**	.824**		.405**	−.327*	.253	.438**	.681**		.480**	−.384**	.408**	.262
negNH2		.832**		.321*				.778**		.506**	−.300*			.752**		.294*		
negNH3	.423**		.845**	−.300*	.281*	.263	.400**		.620**	−.264		.242	.285*		.639**	−.311*	.252	
negNH4		.350*		.629**				.308*		.549**				.25		.555**		
negNH5	.340*				.744**	.411**	.313*			−.265	.438**	.26				−.346*	.511**	
negNH6	.469**			−.24	.472**	.751**	.423**				.266	.604**	.421**				.382**	.311*

negh+1	.818**		.380**	−.261	3475**	.511**	.871**		.411**	−.343*	.382**	.502**	.837**		.372**	−.279*	.460**	.269
negh+2		.838**		.455**				.751**		.613**	−.286*			.748**		.476**		−.237
negh+3	.392**		.855**		.246	.246	.463**		.743**			.342*	.354*		.673**		.239	.346*
negh+4		.319*	−.375**	.639**	−.271	−.269	−.282*	.313*	−.278	.642**		−.264	−.310*	.26	−.302*	.488**		
negh+5	.401**		.282*		.564**	.310*	.410**		.284*		.411**	.333*	.349*				.635**	
negh+6					.275	.603**						.510**				−.299*	.324*	.464**

negh++1	.724**		.338*		.337*	.471**	.760**		.394**	−.347*	.358*	.398**	.784**		.27		.335*	.259
negh++2		.867**		.354*				.718**		.519**				.728**		.433**		
negh++3	.349*		.723**			.275	.394**		.639**			.275			.746**			.336*
negh++4		.256		.561**						.336*				.254		.407**		
negh++5	.267		.251		.562**	.268	.310*		.247	−.257	.448**	.378**	.324*		.257	−.285*	.552**	
negh++6	.276					.456**					−.243	.288*						.477**

*P* < .1; *P < .05; **P < .01.

**Table 5:** Intercorrelations between the reactions to scales of withdrawal from positive and encounter with negative reinforcers of the QDF.

	negNH1	negNH2	negNH3	negNH4	negNH5	negNH6	negh+1	negh+2	negh+3	negh+4	negh+5	negh+6	negh++1	negh++2	negh++3	negh++4	negh++5	negh++6
negNH1	**1**						.840**		.396**	−.246	.455**		.636**		.411**		.353*	
negNH2		**1**						.850**						.826**				
negNH3	.484**		**1**				.380**		.748**	−.413**	.265		.349*		.685**		.271	.245
negNH4	**−**.**278**	.**264**		**1**			−.283*	.394**		.587**	−.266			.290*		.390**		
negNH5	.472**			**−**.**277**	**1**		.314*				.645**		.271				.627**	
negNH6	.477**				.337*	**1**	.472**			−.255	.261	.431**	.424**				.273	.314*

negh+1	.840**		.380**	**−**.283*	.314*	.472**	**1**						.826**		.267		.348*	
negh+2		.850**		.394**				**1**						.829**		.238		
negh+3	.396**		.748**				.428**		**1**				.389**		.626**			
negh+4	−.246		−.413**	.587**		−.255	**−**.345*	.399**	**−**.335*	**1**						.388**		
negh+5	.455**		0.265	**−**.266	.645**	.261	.472**		.301*		**1**		.383**				.688**	
negh+6						.431**					.**276**	**1**	.253				.259	.661**

negh++1	.636**		.349*		.271	.424**	.826**		.389**		.383**	.253	**1**					
negh++2		.826**		.290*				.829**						**1**				
negh++3	.411**		.685**				.267		.626**						**1**			
negh++4				.390**				.238		.388**				.**269**		**1**		
negh++5	.353*		.271		.627**	.273	.348*				.688**	.259	.336*				**1**	
negh++6			.245			.314*						.661**			.345*	**−**.**254**		**1**

*P* < .1; *P < .05; **P < .01.

**Table 6:** Intercorrelations between the reactions to scales of withdrawal from positive and encounter with negative reinforcers of the QDF.

	posH1	posH2	posH3	posH4	posH5	posH6	negH1	negH2	negH3	negH4	negH5	negH6
posH1	**1**						.892**		.426**		.393**	
posH2		**1**						.823**		.328*		
posH3	.529**		**1**				.401**		.815**		.285*	
posH4	−.363**	.585**	−.342*	**1**			−.329*	.581**		.626**	−.297*	−.248
posH5	.524**		.302*	−.297*	**1**		.468**				.654**	
posH6	.499**	−.343*	.478**	−.296*	.309*	**1**	.438**		.424**		.281*	.569**

negH1	.892**		.401**	−.329*	.468**	.438**	**1**					
negH2		.823**		.581**				**1**				
negH3	.426**		.815**			.424**	.362**		**1**			
negH4		.328*		.626**				.340*		**1**		
negH5	.393**		.285*	−.297*	.654**	.281*	.435**				**1**	
negH6				−.248		.569**	**.257**		.279*		**.255**	**1**

*P* < .1; *P < .05; **P < .01.

**Table 7:** Intercorrelations between the reactions to scales of withdrawal from positive and encounter with negative reinforcers of the QDF.

	posNH1	posNH2	posNH3	posNH4	posNH5	posNH6	posh+1	posh+2	posh+3	posh+4	posh+5	posh+6	posh++1	posh++2	posh++3	posh++4	posh++5	posh++6
posH1	.880**		.436**	−.282*	.393**	.494**	.966**		.516**	−.363**	.440**	.527**	.945**		.489**	−.299*	.466**	.329*
posH2		.752**		.379**		−.348*		.932**		.583**	−.342*	−.362**		.935**		.484**		
posH3	.457**		.757**			.287*	.605**		.957**	−.285*	.294*	.420**	.379**		.947**	−.334*	.238	.394**
posH4	−.343*	.545**	−.311*	.738**	−.278	−.412**	−.389**	.521**	−.325*	.900**		−.292*	−.294*	.571**	−.326*	.915**	−.307*	
posH5	.419**		.244		.584**	.319*	.499**		.294*	−.294*	.820**	.476**	.505**	−.24	.280*	−.247	.903**	
posH6	.437**		.419**		.335*	.667**	.476**	−.265	.447**	−.237	.255	.839**	.480**	−.375**	.464**	−.298*	.278	.861**

negH1	.810**		.377**		.429**	.515**	.857**		.421**	−.361*	.388**	.475**	.850**		.339*	−.241	.420**	.276
negH2		.893**		.418**				.766**		.587**	−.255			.771**		.473**		
negH3	.410**		.873**			.290*	.474**		.765**			.341*	.324*		.788**			.378**
negH4		.347*		.723**				.305*		.599**				.308*		.540**		
negH5	.356*		.288*		.612**	.312*	.386**		.287*	−.261	.469**	.389**	.364**		.254	−.277	.641**	
negH6	.269					.589**						.451**				−.289*	.292*	.515**

*P* < .1; *P < .05; **P < .01.

**Table 8:** Intercorrelations between the reactions to scales of withdrawal from positive and encounter with negative reinforcers of the QDF.

	negNH1	negNH2	negNH3	negNH4	negNH5	negNH6	negh+1	negh+2	negh+3	negh+4	negh+5	negh+6	negh++1	negh++2	negh++3	negh++4	negh++5	negh++6
posH1	.795**		.365**		.262	.441**	.895**		.433**	−.308*	.400**		.805**		.338*		.330*	
posH2		.820**		.299*				.803**		.306*				.775**		.236		
posH3	.463**		.661**				.412**		.746**	−.304*	.258		.352*		.725**		.265	
posH4	−.393**	.436**	−.317*	.608**	−.338*	−.241	−.341*	.597**		.619**	−.241	−.280*	−.283*	.522**		.411**	−.299*	
posH5	.393**				.553**	.383**	.492**	−.251			.621**	.305*	.398**				.585**	
posH6	.408**		.265			.532**	.449**	−.248	.405**			.572**	.384**		.360*		.281*	.453**

negH1	.779**		.382**	−.24	.307*	.470**	.961**		.428**	−.305*	.450**	.253	.950**				.358*	
negH2		.875**		.352*				.947**		.298*				.964**		.267		
negH3	.448**		.794**				.383**		.895**	−.298*		.239	.304*		.907**			.276
negH4		.246	−.256	.594**				.389**		.858**				.273	−.251	.806**		−.246
negH5	.434**		.292*		.691**	.291*	.439**		.282*		.901**	.290*	.388**				.935**	
negH6			.25			.415**	.248				.26	.931**	.242		.305*			.889**

*P* < .1; *P < .05; **P < .01.

**Table 9:** Partial correlations of reactions in QDF scales with the personality factors of aggression and depression, controlling for depression and aggression, respectively, (legend see Table 1).

QDF reactions	Aggression (contr. for depression)		Depression (contr. for aggression)	
	posNH	negNH	posNH	negNH
1 (“always happens to me”)	.29*	.27*	.26*	.30*
2 (make the best of it)	.06	−.05	−.02	.12
3 (become angry)	.35**	.28*	.28*	.32*
4 (“such things just happen”)	−.14	−.16	−.07	−.17
5 (blame yourself)	.13	.12	.21	.26*
6 (blame everybody else)	.29*	.26*	.42**	.47**

	posH	negH	posH	negH
1 (“always happens to me”)	.31*	.28*	.26*	.35**
2 (make the best of it)	.09	.08	.09	.04
3 (become angry)	.23	.22	.31*	.39**
4 (“such things just happen”)	−.02	−.06	−.04	−.07
5 (blame yourself)	−.05	.25	.19	.23
6 (blame everybody else)	.19	.10	.15	.17

*P* < .1; *P < .05; **P < .01.

## Appendix

In the following, the male version of the QDF is presented, translated from the original German version. Every situation is labelled by the assigned category in bold letters.

Questionnaire of daily frustrating events.

1. You are sitting in the social room of a bar or restaurant, watching an exciting program on TV. Suddenly, the barkeeper switches off the TV set declaring “it's closing time now” **(posh++)**.
2. You are queuing at a box office of a cinema with the intention to see a movie premiere that you‘ve been waiting for since a long time. Finally, it is your turn, but you are informed that all tickets are sold out **(posNH)**.
3. You did your very best to choose a birthday present for your best friend. While handing over the present, your friend indicates he actually has no use for it **(posh++)**.
4. You have been looking forward very much to a weekend trip with your wife/girl friend/partner. Since a relative of hers/his has become sick and asks her for help, she/he has to cancel the trip **(posh+)**.
5. You are standing in line at a supermarket and you are in a hurry. Suddenly, the customer who has been waiting behind you jumps the queue **(negh++)**.
6. You have ordered a new camera or MP3 player from a mail order company, having been waiting for it for a long time. When it is finally delivered you discover it is defective **(posNH)**.
7. You have made every effort to prepare a pleasant birthday party for your friends. Unfortunately, most of them are in a bad mood and therefore all of them leave the party very early, giving different excuses **(posh++)**.
8. You have completed a very important long letter on your computer. Just as you are going to save the text, your PC breaks down and your information is completely lost **(negNH)**.
9. You are on your way by car to an event that you`ve been looking forward to all day. Totally unexpected you are stuck in a traffic jam **(posNH)**.
10. You are very devoted to your work and try your very best, but your boss does not notice your efforts **(negh+)**.
11. You are standing in line at a supermarket and you are in a hurry. When the customer in front of you wants to pay she realizes that she forgot her purse in the car and first has to fetch it **(negh+)**.
12. For your summer holiday you have booked a hotel room with the view of the sea. After your arrival you notice that outside your window a new high rise building has been constructed and spoils the view **(posNH)**.
13. You took part in a competition and receive a written message that you have won 5.000,00 €! A little later you are informed that accidentally some data have bee mixed up and that, unfortunately, you will not receive anything **(posNH)**.
14. You have very carefully chosen a birthday present for your best friend and you are very anxious to know about his reaction. While you hand over the present your friend, however, he states that he had already got the same object **(posh+)**.
15. You have an appointment with your mechanic who has to look after a blocked drain in your flat. When he does not turn up and you finally reach him by phone he declares, that he first had to perform some other orders more important than this triviality **(negh++)**.
16. Just for a moment, you are leaving your flat without taking a key with you while the door remains open. But a heavy blast shuts the door and you are locked out **(negNH)**.
17. You are preparing a sophisticated meal while you receive a telephone call from a friend. You are so preoccupied with your conversation that in the kitchen the food is burning **(negh+)**.
18. You have prepared your birthday party with great devotion and effort and are looking forward to the arrival of your friends who live in a different place far away. A thunderstorm makes it impossible for them to come because the streets are impassable and the railroad traffic is interrupted **(posh+)**.
19. You have asked your neighbour to help you painting the living room at the weekend. When he does not appear and you call him later that evening your neighbour explains that he had family obligations and therefore did not have time to help **(negh+)**.
20. At work you always give your very best and you are also very conscientious. Yet, your boss always criticizes you for working too slowly or making too many mistakes **(negh++)**.
21. You are comfortably sitting in front of your TV watching an exciting movie. Suddenly, some of your relatives on their way through are ringing the bell at the front door **(posh+)**.
22. You`re on your way to an important meeting. But on the roadside passing cars splash your elegant clothes **(negNH)**.
23. You are in a hurry while you`re shopping. Just as you want to pay, a technical problem occurs at the cash desk so that you have to wait even longer **(negNH)**.
24. You`re just preparing a sophisticated meal when all of a sudden a newspaper agent rings the door bell to sell you a subscription. Although you explain that you`re just occupied with cooking, you cannot get rid of him and the food is burning **(negh++)**.
25. Late in the evening you return home extremely hungry but you do not find anything to eat in the refrigerator. When you decide to go to the snack bar next door, it has just closed **(posNH)**.
26. You want to empty your trash bag into the large garbage container. On your way, the trash bag rips open and your garbage is scattered allover the entrance hall **(negNH)**.
27. You`re already late on your way to work. When you arrive at the bus stop you just see the last bus leaving **(negNH)**.
28. You`re getting home very tired, looking forward to your well-earned rest. After you have just collapsed into bed in an exhausted state the phone starts ringing loudly **(posNH)**.
29. Within the previous days a large amount of laundry has accumulated. As you finally succeed in activating the washing machine you recognize that it is out of order **(negNH)**.
30. You are on your way to an important appointment by car. Unexpectedly, there`s a new detour of 10 km on your route so that you arrive too late **(negNH)**.
31. In the evening you are sitting in front of your TV, watching an exciting movie. Suddenly there`s a screen failure **(posNH)**.
32. You have an appointment with your wife/girl friend/partner for a weekend trip and you`re looking forward to it very much. When already waiting for her/him, she/he rings you up and tells you that she/he cannot meet you because she/he rather decided to go to a musical with others on that weekend **(posh++)**.
